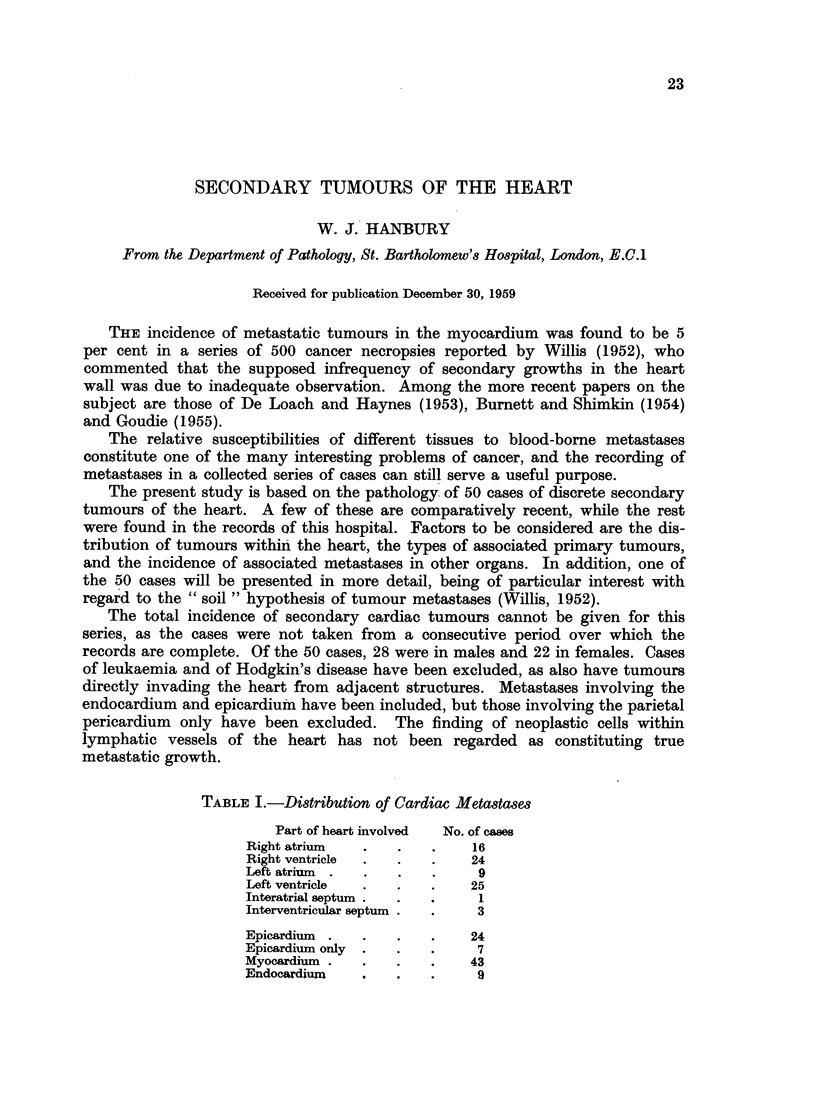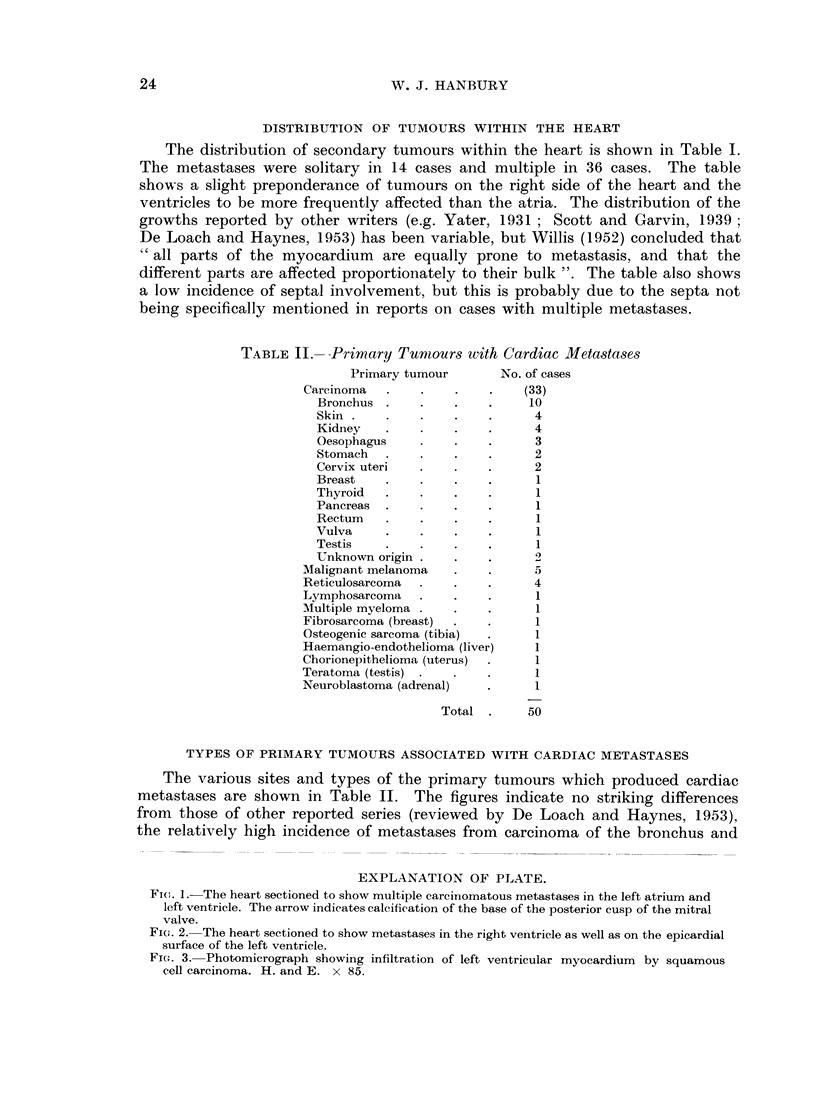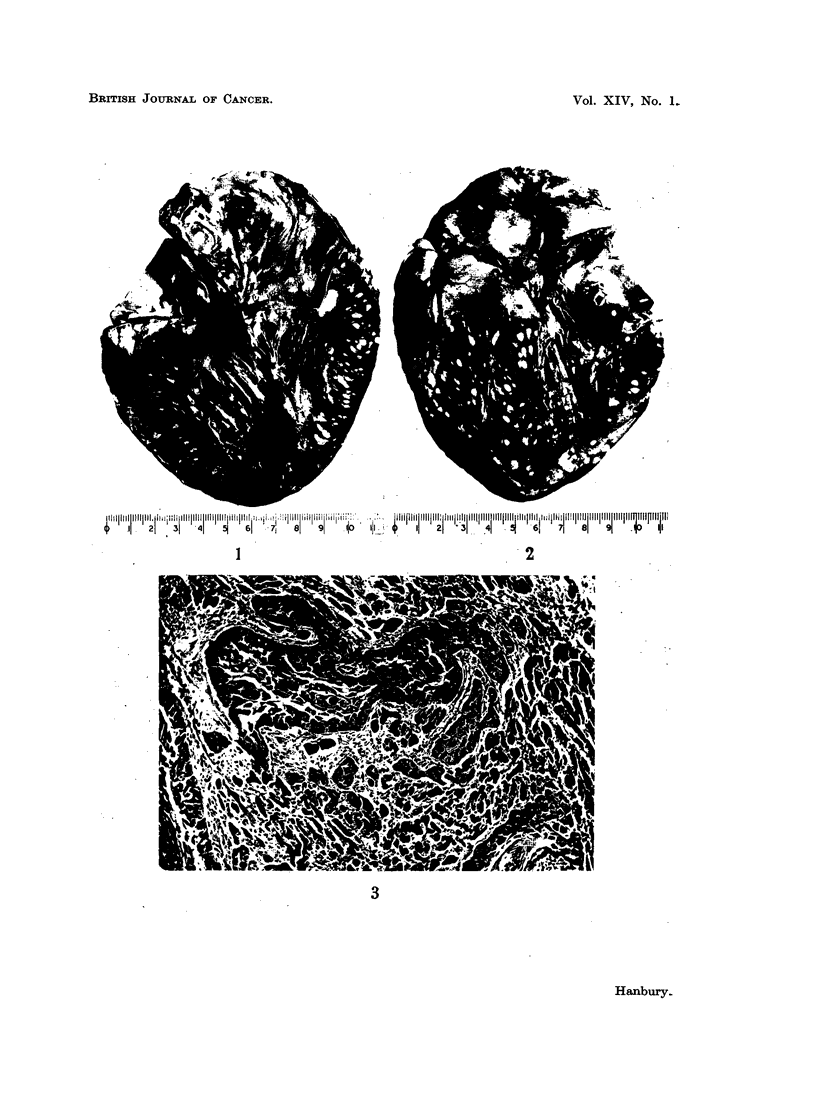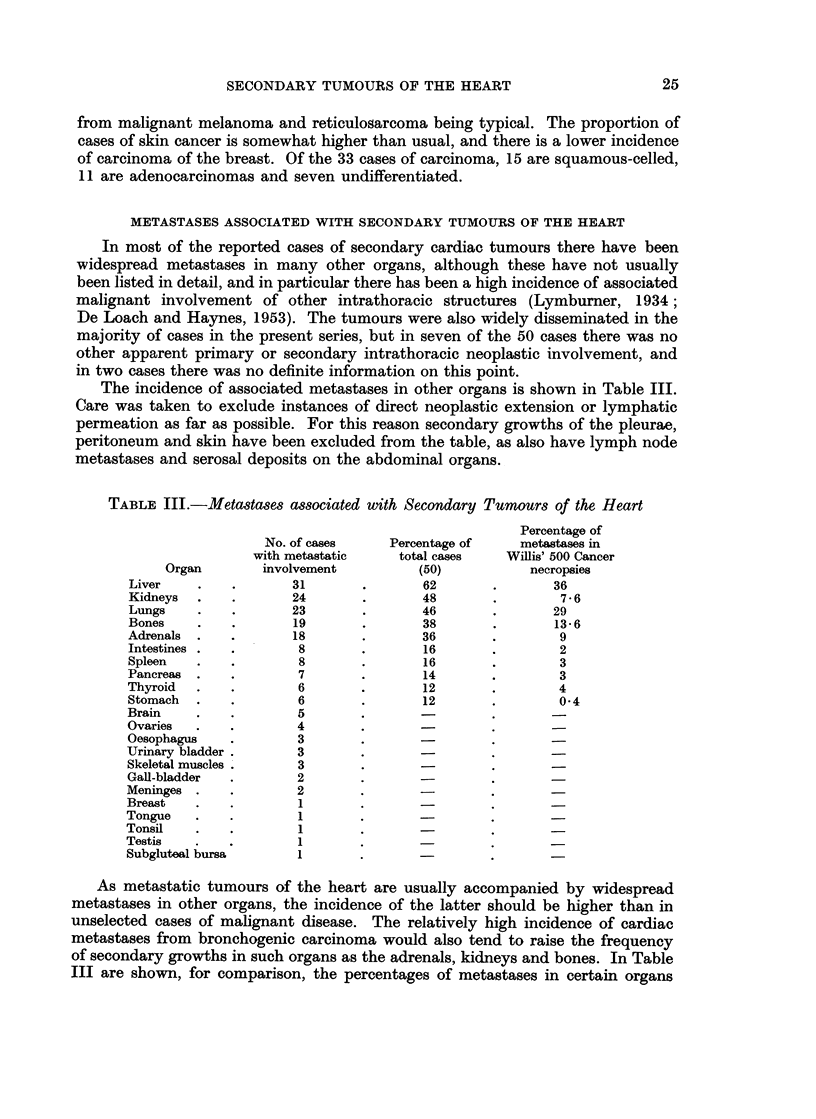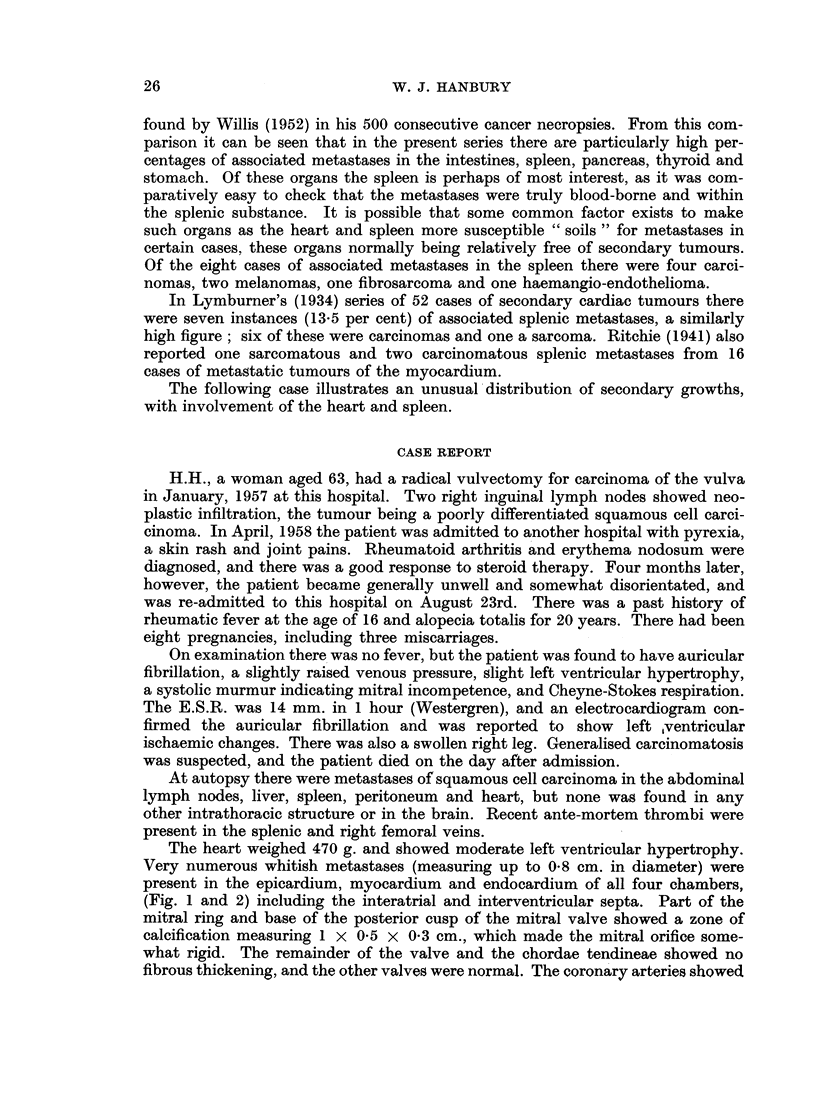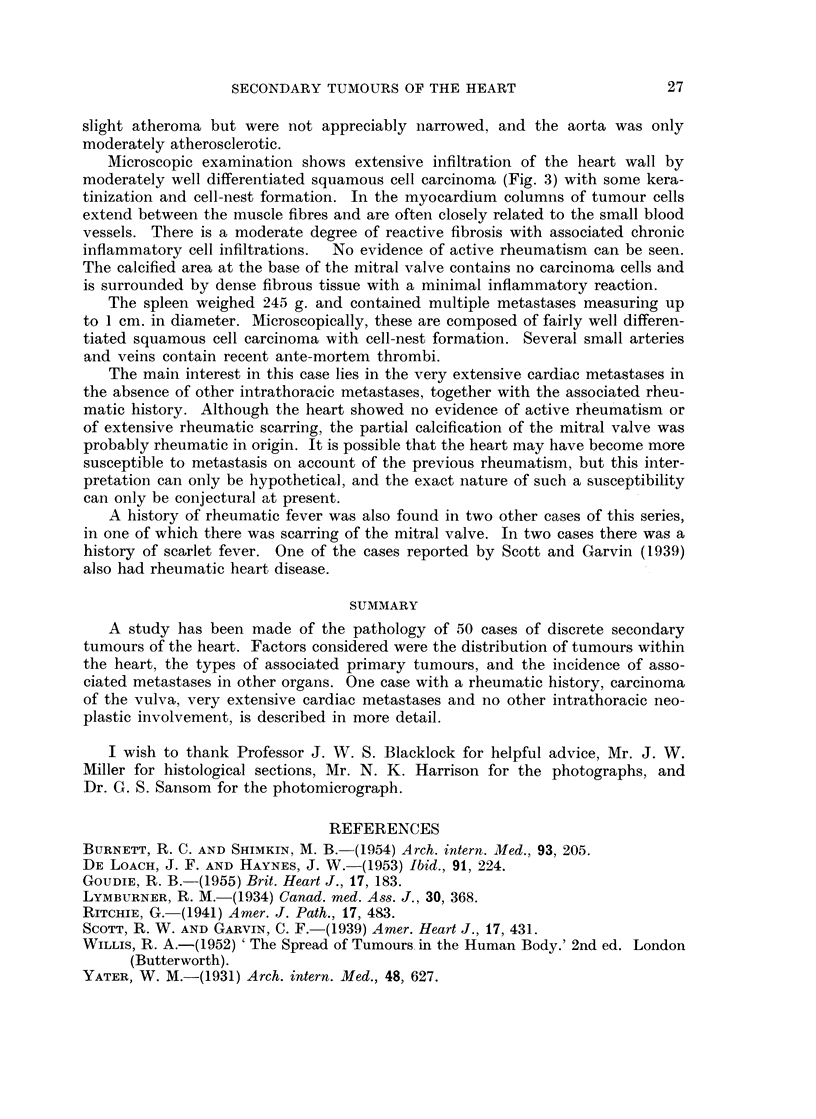# Secondary Tumours of the Heart

**DOI:** 10.1038/bjc.1960.3

**Published:** 1960-03

**Authors:** W. J. Hanbury

## Abstract

**Images:**


### SECONDARY TUMOURS OF THE HEART

W. J. HANBURY

*From the Department of Pathology, St. Bartholomew's Hospital, London, E.C.*1

Received for publication December 30, 1959

THE incidence of metastatic tumours in the myocardium was found to be 5 per cent in a series of 500 cancer necropsies reported by Willis (1952), who commented that the supposed infrequency of secondary growths in the heart wall was due to inadequate observation. Among the more recent papers on the subject are those of De Loach and Haynes (1953), Burnett and Shimkin (1954) and Goudie (1955).

The relative susceptibilities of different tissues to blood-borne metastases constitute one of the many interesting problems of cancer, and the recording of metastases in a collected series of cases can still serve a useful purpose.

The present study is based on the pathology of 50 cases of discrete secondary tumours of the heart. A few of these are comparatively recent, while the rest were found in the records of this hospital. Factors to be considered are the distribution of tumours within the heart, the types of associated primary tumours, and the incidence of associated metastases in other organs. In addition, one of the 50 cases will be presented in more detail, being of particular interest with regard to the " soil " hypothesis of tumour metastases (Willis, 1952).

The total incidence of secondary cardiac tumours cannot be given for this series, as the cases were not taken from a consecutive period over which the records are complete. Of the 50 cases, 28 were in males and 22 in females. Cases of leukaemia and of Hodgkin's disease have been excluded, as also have tumours directly invading the heart from adjacent structures. Metastases involving the endocardium and epicardium have been included, but those involving the parietal pericardium only have been excluded. The finding of neoplastic cells within lymphatic vessels of the heart has not been regarded as constituting true metastatic growth.

TABLE I.—*Distribution of Cardiac Metastases*

| Part of heart involved | No. of cases |
|---|---|
| Right atrium | 16 |
| Right ventricle | 24 |
| Left atrium | 9 |
| Left ventricle | 25 |
| Interatrial septum | 1 |
| Interventricular septum | 3 |
| | |
| Epicardium | 24 |
| Epicardium only | 7 |
| Myocardium | 43 |
| Endocardium | 9 |

### DISTRIBUTION OF TUMOURS WITHIN THE HEART

The distribution of secondary tumours within the heart is shown in Table I. The metastases were solitary in 14 cases and multiple in 36 cases. The table shows a slight preponderance of tumours on the right side of the heart and the ventricles to be more frequently affected than the atria. The distribution of the growths reported by other writers (e.g. Yater, 1931 ; Scott and Garvin, 1939 ; De Loach and Haynes, 1953) has been variable, but Willis (1952) concluded that "all parts of the myocardium are equally prone to metastasis, and that the different parts are affected proportionately to their bulk ". The table also shows a low incidence of septal involvement, but this is probably due to the septa not being specifically mentioned in reports on cases with multiple metastases.

TABLE II.—*Primary Tumours with Cardiac Metastases*

| Primary tumour | No. of cases |
|---|---|
| Carcinoma . . . . | (33) |
| Bronchus . . . . | 10 |
| Skin . . . . . | 4 |
| Kidney . . . . | 4 |
| Oesophagus . . . | 3 |
| Stomach . . . . | 2 |
| Cervix uteri . . . | 2 |
| Breast . . . . | 1 |
| Thyroid . . . . | 1 |
| Pancreas . . . . | 1 |
| Rectum . . . . | 1 |
| Vulva . . . . | 1 |
| Testis . . . . | 1 |
| Unknown origin . . . | 2 |
| Malignant melanoma . . | 5 |
| Reticulosarcoma . . . | 4 |
| Lymphosarcoma . . . | 1 |
| Multiple myeloma . . . | 1 |
| Fibrosarcoma (breast) . . | 1 |
| Osteogenic sarcoma (tibia) . | 1 |
| Haemangio-endothelioma (liver) | 1 |
| Chorionepithelioma (uterus) . | 1 |
| Teratoma (testis) . . . | 1 |
| Neuroblastoma (adrenal) . | 1 |
| Total . | 50 |

### TYPES OF PRIMARY TUMOURS ASSOCIATED WITH CARDIAC METASTASES

The various sites and types of the primary tumours which produced cardiac metastases are shown in Table II. The figures indicate no striking differences from those of other reported series (reviewed by De Loach and Haynes, 1953), the relatively high incidence of metastases from carcinoma of the bronchus and

EXPLANATION OF PLATE.

FIG. 1.—The heart sectioned to show multiple carcinomatous metastases in the left atrium and left ventricle. The arrow indicates calcification of the base of the posterior cusp of the mitral valve.

FIG. 2.—The heart sectioned to show metastases in the right ventricle as well as on the epicardial surface of the left ventricle.

FIG. 3.—Photomicrograph showing infiltration of left ventricular myocardium by squamous cell carcinoma. H. and E. × 85.

1

2

3

from malignant melanoma and reticulosarcoma being typical. The proportion of cases of skin cancer is somewhat higher than usual, and there is a lower incidence of carcinoma of the breast. Of the 33 cases of carcinoma, 15 are squamous-celled, 11 are adenocarcinomas and seven undifferentiated.

### METASTASES ASSOCIATED WITH SECONDARY TUMOURS OF THE HEART

In most of the reported cases of secondary cardiac tumours there have been widespread metastases in many other organs, although these have not usually been listed in detail, and in particular there has been a high incidence of associated malignant involvement of other intrathoracic structures (Lymburner, 1934; De Loach and Haynes, 1953). The tumours were also widely disseminated in the majority of cases in the present series, but in seven of the 50 cases there was no other apparent primary or secondary intrathoracic neoplastic involvement, and in two cases there was no definite information on this point.

The incidence of associated metastases in other organs is shown in Table III. Care was taken to exclude instances of direct neoplastic extension or lymphatic permeation as far as possible. For this reason secondary growths of the pleurae, peritoneum and skin have been excluded from the table, as also have lymph node metastases and serosal deposits on the abdominal organs.

TABLE III.—*Metastases associated with Secondary Tumours of the Heart*

| Organ | No. of cases with metastatic involvement | Percentage of total cases (50) | Percentage of metastases in Willis' 500 Cancer necropsies |
|---|---|---|---|
| Liver | 31 | 62 | 36 |
| Kidneys | 24 | 48 | 7·6 |
| Lungs | 23 | 46 | 29 |
| Bones | 19 | 38 | 13·6 |
| Adrenals | 18 | 36 | 9 |
| Intestines | 8 | 16 | 2 |
| Spleen | 8 | 16 | 3 |
| Pancreas | 7 | 14 | 3 |
| Thyroid | 6 | 12 | 4 |
| Stomach | 6 | 12 | 0·4 |
| Brain | 5 | — | — |
| Ovaries | 4 | — | — |
| Oesophagus | 3 | — | — |
| Urinary bladder | 3 | — | — |
| Skeletal muscles | 3 | — | — |
| Gall-bladder | 2 | — | — |
| Meninges | 2 | — | — |
| Breast | 1 | — | — |
| Tongue | 1 | — | — |
| Tonsil | 1 | — | — |
| Testis | 1 | — | — |
| Subgluteal bursa | 1 | — | — |

As metastatic tumours of the heart are usually accompanied by widespread metastases in other organs, the incidence of the latter should be higher than in unselected cases of malignant disease. The relatively high incidence of cardiac metastases from bronchogenic carcinoma would also tend to raise the frequency of secondary growths in such organs as the adrenals, kidneys and bones. In Table III are shown, for comparison, the percentages of metastases in certain organs found by Willis (1952) in his 500 consecutive cancer necropsies. From this comparison it can be seen that in the present series there are particularly high percentages of associated metastases in the intestines, spleen, pancreas, thyroid and stomach. Of these organs the spleen is perhaps of most interest, as it was comparatively easy to check that the metastases were truly blood-borne and within the splenic substance. It is possible that some common factor exists to make such organs as the heart and spleen more susceptible " soils " for metastases in certain cases, these organs normally being relatively free of secondary tumours. Of the eight cases of associated metastases in the spleen there were four carcinomas, two melanomas, one fibrosarcoma and one haemangio-endothelioma.

In Lymburner's (1934) series of 52 cases of secondary cardiac tumours there were seven instances (13·5 per cent) of associated splenic metastases, a similarly high figure ; six of these were carcinomas and one a sarcoma. Ritchie (1941) also reported one sarcomatous and two carcinomatous splenic metastases from 16 cases of metastatic tumours of the myocardium.

The following case illustrates an unusual distribution of secondary growths, with involvement of the heart and spleen.

### CASE REPORT

H.H., a woman aged 63, had a radical vulvectomy for carcinoma of the vulva in January, 1957 at this hospital. Two right inguinal lymph nodes showed neoplastic infiltration, the tumour being a poorly differentiated squamous cell carcinoma. In April, 1958 the patient was admitted to another hospital with pyrexia, a skin rash and joint pains. Rheumatoid arthritis and erythema nodosum were diagnosed, and there was a good response to steroid therapy. Four months later, however, the patient became generally unwell and somewhat disorientated, and was re-admitted to this hospital on August 23rd. There was a past history of rheumatic fever at the age of 16 and alopecia totalis for 20 years. There had been eight pregnancies, including three miscarriages.

On examination there was no fever, but the patient was found to have auricular fibrillation, a slightly raised venous pressure, slight left ventricular hypertrophy, a systolic murmur indicating mitral incompetence, and Cheyne-Stokes respiration. The E.S.R. was 14 mm. in 1 hour (Westergren), and an electrocardiogram confirmed the auricular fibrillation and was reported to show left ventricular ischaemic changes. There was also a swollen right leg. Generalised carcinomatosis was suspected, and the patient died on the day after admission.

At autopsy there were metastases of squamous cell carcinoma in the abdominal lymph nodes, liver, spleen, peritoneum and heart, but none was found in any other intrathoracic structure or in the brain. Recent ante-mortem thrombi were present in the splenic and right femoral veins.

The heart weighed 470 g. and showed moderate left ventricular hypertrophy. Very numerous whitish metastases (measuring up to 0·8 cm. in diameter) were present in the epicardium, myocardium and endocardium of all four chambers, (Fig. 1 and 2) including the interatrial and interventricular septa. Part of the mitral ring and base of the posterior cusp of the mitral valve showed a zone of calcification measuring 1 × 0·5 × 0·3 cm., which made the mitral orifice somewhat rigid. The remainder of the valve and the chordae tendineae showed no fibrous thickening, and the other valves were normal. The coronary arteries showed slight atheroma but were not appreciably narrowed, and the aorta was only moderately atherosclerotic.

Microscopic examination shows extensive infiltration of the heart wall by moderately well differentiated squamous cell carcinoma (Fig. 3) with some keratinization and cell-nest formation. In the myocardium columns of tumour cells extend between the muscle fibres and are often closely related to the small blood vessels. There is a moderate degree of reactive fibrosis with associated chronic inflammatory cell infiltrations. No evidence of active rheumatism can be seen. The calcified area at the base of the mitral valve contains no carcinoma cells and is surrounded by dense fibrous tissue with a minimal inflammatory reaction.

The spleen weighed 245 g. and contained multiple metastases measuring up to 1 cm. in diameter. Microscopically, these are composed of fairly well differentiated squamous cell carcinoma with cell-nest formation. Several small arteries and veins contain recent ante-mortem thrombi.

The main interest in this case lies in the very extensive cardiac metastases in the absence of other intrathoracic metastases, together with the associated rheumatic history. Although the heart showed no evidence of active rheumatism or of extensive rheumatic scarring, the partial calcification of the mitral valve was probably rheumatic in origin. It is possible that the heart may have become more susceptible to metastasis on account of the previous rheumatism, but this interpretation can only be hypothetical, and the exact nature of such a susceptibility can only be conjectural at present.

A history of rheumatic fever was also found in two other cases of this series, in one of which there was scarring of the mitral valve. In two cases there was a history of scarlet fever. One of the cases reported by Scott and Garvin (1939) also had rheumatic heart disease.

### SUMMARY

A study has been made of the pathology of 50 cases of discrete secondary tumours of the heart. Factors considered were the distribution of tumours within the heart, the types of associated primary tumours, and the incidence of associated metastases in other organs. One case with a rheumatic history, carcinoma of the vulva, very extensive cardiac metastases and no other intrathoracic neoplastic involvement, is described in more detail.

I wish to thank Professor J. W. S. Blacklock for helpful advice, Mr. J. W. Miller for histological sections, Mr. N. K. Harrison for the photographs, and Dr. G. S. Sansom for the photomicrograph.

### REFERENCES

Burnett, R. C. and Shimkin, M. B.—(1954) *Arch. intern. Med.*, **93**, 205.
De Loach, J. F. and Haynes, J. W.—(1953) *Ibid.*, **91**, 224.
Goudie, R. B.—(1955) *Brit. Heart J.*, **17**, 183.
Lymburner, R. M.—(1934) *Canad. med. Ass. J.*, **30**, 368.
Ritchie, G.—(1941) *Amer. J. Path.*, **17**, 483.
Scott, R. W. and Garvin, C. F.—(1939) *Amer. Heart J.*, **17**, 431.
Willis, R. A.—(1952) ' The Spread of Tumours in the Human Body.' 2nd ed. London (Butterworth).
Yater, W. M.—(1931) *Arch. intern. Med.*, **48**, 627.